# Induction of Programmed Cell Death in *Acanthamoeba culbertsoni* by the Repurposed Compound Nitroxoline

**DOI:** 10.3390/antiox12122081

**Published:** 2023-12-06

**Authors:** Rubén L. Rodríguez-Expósito, Ines Sifaoui, María Reyes-Batlle, Frieder Fuchs, Patrick L. Scheid, José E. Piñero, Robert Sutak, Jacob Lorenzo-Morales

**Affiliations:** 1 Instituto Universitario de Enfermedades Tropicales y Salud Pública de Canarias (IUETSPC), Universidad de La Laguna (ULL), Avda. Astrofísico Fco. Sánchez, S/N, 38203 San Cristóbal de La Laguna, Spain; rrodrige@ull.edu.es (R.L.R.-E.); isifaoui@ull.edu.es (I.S.); mreyesba@ull.edu.es (M.R.-B.); 2 Departamento de Obstetricia y Ginecología, Pediatría, Medicina Preventiva y Salud Pública, Toxicología, Medicina Legal y Forense y Parasitología, Universidad de La Laguna, 38203 San Cristóbal de La Laguna, Spain; 3 Institute for Medical Microbiology, Immunology and Hygiene, University Hospital Cologne, Faculty of Medicine, University of Cologne, 50935 Cologne, Germany; frieder.fuchs@uk-koeln.de; 4 Department of Microbiology and Hospital Hygiene, Bundeswehr Central Hospital Koblenz, 56072 Koblenz, Germany; 5 Parasitology Lab., Central Military Hospital Koblenz, 56072 Koblenz, Germany; 6 Department of Biology, Working Group Parasitology and Infection Biology, University Koblenz, 56070 Koblenz, Germany; 7 Centro de Investigación Biomédica en Red de Enfermedades Infecciosas (CIBERINFEC), Instituto de Salud Carlos III, 28220 Madrid, Spain; 8 Department of Parasitology, Faculty of Science, Charles University, BIOCEV, 252 50 Vestec, Czech Republic

**Keywords:** *Acanthamoeba*, nitroxoline, programmed cell death, autophagy, cytoskeleton, proteomic analysis

## Abstract

*Acanthamoeba* is a ubiquitous genus of amoebae that can act as opportunistic parasites in both humans and animals, causing a variety of ocular, nervous and dermal pathologies. Despite advances in *Acanthamoeba* therapy, the management of patients with *Acanthamoeba* infections remains a challenge for health services. Therefore, there is a need to search for new active substances against Acanthamoebae. In the present study, we evaluated the amoebicidal activity of nitroxoline against the trophozoite and cyst stages of six different strains of *Acanthamoeba*. The strain *A. griffini* showed the lowest IC_50_ value in the trophozoite stage (0.69 ± 0.01 µM), while the strain *A. castellanii* L-10 showed the lowest IC_50_ value in the cyst stage (0.11 ± 0.03 µM). In addition, nitroxoline induced in treated trophozoites of *A. culbertsoni* features compatibles with apoptosis and autophagy pathways, including chromatin condensation, mitochondrial malfunction, oxidative stress, changes in cell permeability and the formation of autophagic vacuoles. Furthermore, proteomic analysis of the effect of nitroxoline on trophozoites revealed that this antibiotic induced the overexpression and the downregulation of proteins involved in the apoptotic process and in metabolic and biosynthesis pathways.

## 1. Introduction

Acanthamoebae are among the most commonly isolated free-living amoebae (FLA) in the environment and clinical samples. Their life cycle includes a vegetative form, or trophozoite, which is a motile trophic stage, when the amoeba is in a humid, favorable and nutrition-rich environment. Trophozoites may form a resistant and dormant cyst as soon as they are exposed to adverse conditions. Due to the ubiquitous nature of the *Acanthamoeba* genus in the environment, people may frequently come in contact with amoebae. Amoebae of the genus *Acanthamoeba* have been isolated from diverse natural or artificial sources including sea water, soil, air, lakes, bottled mineral and tap water, air-conditioning units, contact lenses and their cases or even hospital facilities [1,2,3].

Acanthamoebae may act as a parasite in diverse infections in animals and humans. Several strains of the genus *Acanthamoeba* are the etiological agents of the multifocal encephalitis known as granulomatous amoebic encephalitis (GAE) [4,5,6]. Whereas the dangerous, subacute GAE is limited to immunocompromised hosts, *Acanthamoeba* keratitis (AK) more frequently affects immunocompetent hosts. This ocular infection is a progressive disease that primarily affects soft-contact-lens wearers. Its late diagnosis or ineffective treatment can lead to complete loss of vision in patients with this severe corneal infection [3,7,8,9]. In addition, *Acanthamoeba* has also been reported as a cause of rare opportunistic skin infections and pneumonitis [10,11,12,13].

In addition to their role as human pathogens, *Acanthamoeba* have received particular attention due to the fact that they can act as vectors and reservoirs for diverse microorganisms [14,15,16]. Most environmental or clinical isolates of *Acanthamoeba* associated with AK proved to harbor some endocytobionts, including different species of pathogenic bacteria resistant to antibiotics, such as members of the genus *Legionella*, *Escherichia coli*, *Mycobacterium avium*, *Streptococcus* spp., *Staphylococcus aureus*, *Ricketsiales* and *Chlamydiales*, yeasts including *Cryptococcus neoformans*, *Histoplasma capsulatum* and *Blastomyces dermatitides* and viruses like adenoviruses, pandoviruses or mimiviruses [14,16,17,18].

Acanthamoebae are highly resistant to compounds used as disinfectants or medical treatments for infections caused by these protozoa. Targeting both the vegetative trophozoite and the cyst resistant forms of these amoebae is important both for the treatment of the infections caused by this parasite and for the elimination of other pathogens associated with it intracellularly [3,7,8,19]. Currently, despite several compounds or combination being described for the treatment of *Acanthamoeba* infections, no single agent has been shown to be effective against both the trophozoite and the cyst forms of Acanthamoebae [3,7,8]. Additionally, treatment strategies using a combination of different drugs are often effective in the early stages of infection, but often ineffective after prolonged application against chronic infections, mainly due to the development of amoebic resistance, toxicity or side effects [19,20,21,22].

In recent years, drug repurposing has attracted pharmaceutical research because of the option to use approved or investigational drugs, which have been applied to treat other diseases, in a new therapeutic area that requires new drug candidates, while reducing costly and time-consuming pharmacokinetic and toxicity testing [21,23,24,25].

**Figure 1:**
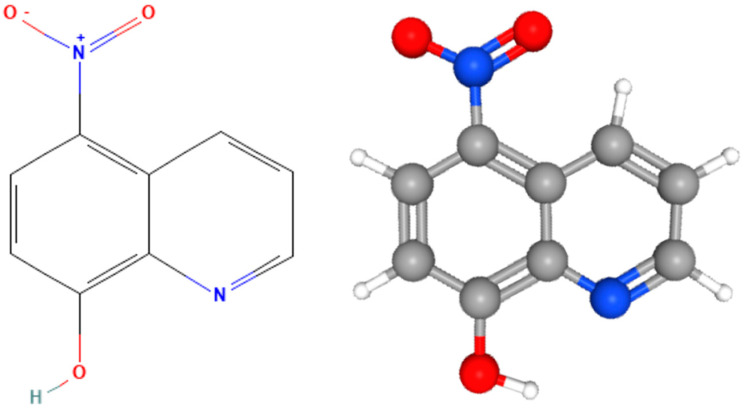
Chemical structure of nitroxoline (5−nitro−8-hydroxyquinoline). C_9_H_6_N_2_O_3_ (molecular weight: 190.16 g/mL).

Nitroxoline, a hydroxyquinoline derivate, has been used for many years to treat urinary tract infections (UTIs) in Europe [26,27] (Figure 1). Nitroxolineclinical efficacy and safety were confirmed in a multicenter study on cystitis treatment [28]. This drug is known for its antibacterial, amoebicidal, antifungal, antiviral, anticancer and anti-inflammatory activities [29,30,31,32,33,34,35,36]. In addition, nitroxoline was approved for use as an anti-neurodegenerative drug to treat Alzheimer’s disease and cancer in humans [37,38,39,40,41]. Laurie et al. (2018) demonstrated that nitroxoline showed amoebicidal activity against the pathogen *Balamuthia mandrillaris* [31]. Nitroxoline was the most potent inhibitor of *B. mandrillaris*, with an IC_50_ of 2.84 µM for trophozoites and of 15.48 µM for cysts. Moreover, Spottiswoode et al. (2023) reported a clinical case of a patient with *B. mandrillaris* granulomatous amebic encephalitis who survived after receiving treatment with nitroxoline [42].

In a previous study, we reported that nitroxoline showed promising potency and selectivity in *Naegleria fowleri* inhibition, which led us to demonstrate that this compound triggered events compatible with apoptotic and autophagic programmed cell death processes in *N. fowleri* [43]. In the present research, we evaluated the efficacy of nitroxoline against six different strains of *Acanthamoeba*, focusing our efforts on further investigating its novel amoebicidal activity. To the best of our knowledge, this is the first time that the activity of nitroxoline against *Acanthamoeba* has been investigated.

## 2. Materials and Methods

### 2.1. Molecules

The compound nitroxoline was afforded by Rosen Pharma St. Ingbert Germany. The stock solution and dilutions were made following the protocol previously described by Chao-Pellicer et al. (2023) [43].

### 2.2. Acanthamoeba *spp.* Strains

The in vitro assays for the evaluation of the antiamoebic effect of the molecule nitroxoline were performed using six strains of *Acanthamoeba*: *Acanthamoeba castellanii* Neff, genotype T4, obtained from the American Type Culture Collection (LG Promochem, Barcelona, Spain), (ATCC^®^ 30010™), *Acanthamoeba polyphaga*, genotype T4 (ATCC^®^ 30461™), *Acanthamoeba griffini*, genotype T3, obtained according to a previous study [44], *Acanthamoeba quina*, genotype T4 (ATCC^®^ 50241™), *Acanthamoeba castellanii* L-10, genotype T4, isolated according to a previous study [45] and *Acanthamoeba culbertsoni*, genotype T10 (ATCC 30171). These *Acanthamoeba* strains were grown axenically in Peptone Yeast Glucose (PYG) medium (0.75% (*w*/*v*) proteose peptone, 0.75% (*w*/*v*) yeast extract and 1.5% (*w*/*v*) glucose), containing 40 µg of gentamicin mL^−1^ (Biowest, Nuaillé, France).

### 2.3. In Vitro Effect against the Trophozoite Stage

The amoebicidal in vitro activity of nitroxoline against the trophozoite stage of six different *Acanthamoeba* strains was determined according to the alamarBlue™ reagent method (Life Technologies, Madrid, Spain) after 96 h of treatment, as previously described [46]. Briefly, the cells were seeded in 96-well plates and treated with different drug dilutions. The emitted fluorescence by the alamarBlue™ reagent was measured using an EnSpire^®^ Multimode Plate Reader (Perkin Elmer, Madrid, Spain) to determine the IC_50_ and IC_90_ values of nitroxoline.

### 2.4. In Vitro Effect against the Cyst Stage

To carry out this assay, the cysts of the six tested strains of *Acanthamoeba* were prepared as described before [46]. The cysticidal activity (IC_50_) was evaluated by the alamarBlue™ method after 168 h of treatment, following the protocol defined for the trophozoite assay [46].

### 2.5. Evaluation of Actin Distribution

Trophozoites of *Acanthamoeba culbertsoni* were treated first with the IC_90_ of nitroxoline. After 24 h of incubation, the cells were prepared and treated with phalloidin-tetramethylrhodamine B isothiocynate (phalloidin-TRITC; Sigma-Aldrich, Madrid, Spain), following a protocol previously described [46]. Finally, the cells were examined by Z-stack imaging using the inverted-light confocal microscope Leica DMI 4000 B with a 63× objective (Leica Microsystems, Germany) at λ_exc_ = 540 nm and λ_em_ = 570 nm.

### 2.6. Immunofluorescence Staining of Intracellular Tubulin in Acanthamoeba culbertsoni

The trophozoites of *Acanthamoeba culbertsoni* were incubated following the same method described for the fluorescent staining of intracellular actin. Then, the cells were treated and incubated with an anti-tubulin antibody (monoclonal anti-α-tubulin antibody produced in mouse, Sigma-Aldrich, Madrid, Spain). Next, the cells were incubated with the goat anti-mouse IgG (H+L) Highly Cross-Adsorbed Secondary Antibody, Alexa Fluor Plus 594 (Thermo Fisher Scientific, Rockford, IL, USA), following the method reported in a previous study [46]. Finally, the cells with treated nitroxoline and untreated trophozoites (negative control) were examined following the method described for the actin distribution assay at λ_exc_ = 590 nm and λ_em_ = 617 nm.

### 2.7. In Vitro Labelling of Autophagic Vacuoles in Acanthamoeba culbertsoni

The monodansylcadaverine (MDC) autofluorescent dye has been shown to accumulate in acidic autophagic vacuoles. The concentration of this compound in autophagic vacuoles is the consequence of the combination of ion trapping and specific interactions with vesicle membrane lipids. This assay was carried out by following the method described in a previous study [47]. Finally, the trophozoites were observed using an inverted microscope EVOS™ FL Cell Imaging System M5000 (Life Technologies, USA).

### 2.8. Proteomic Analysis of the Effect of Nitroxoline in Acanthamoeba Castellanii L-10 Trophozoites

#### 2.8.1. Comparative Label-Free Proteomics

The proteomic analysis of the effect of nitroxoline was performed based on a previous study by Arbon et al. (2022) [48]. The concentration of *Acanthamoeba castellanii* L-10 trophozoites used was 10^6^ cells/mL. The cells were treated with the determined IC_50_ value of nitroxoline for 24 h and washed 3 times with PBS 1x; then, the cell pellets were subjected to further processing. Untreated cells were prepared as a control group. Both groups were prepared in three biological replicates.

#### 2.8.2. Protein Digestion

Untreated and treated cells were homogenized, boiled at 95 °C for 10 min until lysed, using 100 mM triethylammonium bicarbonate containing 2% sodium deoxycholate, 40 mM chloroacetamide and 10 mM TCEP (tris(2-carboxyethyl)phosphine), and later sonicated (Bandelin Sonoplus Mini 20, MS 1.5). For the MS analysis, samples with a protein concentration of 30 µg were used.

The obtained proteins were further handled using SP3 beads according to Hughes et al. (2019) [49]. Briefly, 5 µL of SP3 beads was added to the prepared protein samples dissolved in buffer lysis and brought to the volume of 50 µL with 100 mM TEAB. Protein binding was obtained by adding ethanol up to a final concentration of 60% (*v*/*v*). After 5 min at RT and with the aid of a magnetic rack, the unbound supernatant was eliminated from the tubes. Before digestion, the beads were washed twice with 180 µL of 80% ethanol. The samples were digested overnight at 37 °C using trypsin (trypsin/protein ratio 1/30) reconstituted in 100 mM TEAB. Finally, the samples were acidified with TFA to 1% final concentration, and the peptides were desalted using in-house-made stage tips packed with C18 disks (Empore), according to Rappsilber et al. (2007) [50].

#### 2.8.3. nLC-MS 2 Analysis

For the LC/MS analysis, a nano reversed-phase column (EASY-Spray column, 50 cm × 75 µm ID, PepMap C18, 2 µm particles, 100 Å pore size) was used. The samples were loaded onto the trap column (C18 PepMap100, 5 μm particle size, 300 μm × 5 mm, Thermo Scientific) for 4 min in loading buffer (water, 2% acetonitrile and 0.1% trifluoroacetic acid) at 18 μL/min. A mobile phase consisting of formic acid at 0.1% (eluent A) and acetonitrile containing 0.1% formic acid (eluent B) was used. The peptides were eluted with a gradient of mobile phase B from 4% to 35% in 120 min. The peptides cations generated via electrospray ionization were analyzed on a Thermo Orbitrap Fusion mass spectrometer (Q-OT-qIT, Thermo Scientific). The data were obtained using the Orbitrap analyzer, at a resolution of 120 K (at 200 *m*/*z*) with a 5 × 10^5^ ion count target in a scan range from 350 to 1400 *m*/*z*. Tandem MS was conducted by isolation at 1.5 Th with the quadrupole, HCD fragmentation with normalized collision energy of 30 eV and rapid-scanning MS analysis in the ion trap. The MS^2^ ion count target was set to 10^4^, and the max injection time was 35 ms. Precursors with a charge state of 2–6 were exclusively sampled for MS^2^. The duration of dynamic exclusion was fixed to 30 s, while a 10 ppm tolerance around the selected precursor and its isotopes was set. Monoisotopic precursor selection was turned on. The instrument was run in top speed mode with 2 s cycles [51].

#### 2.8.4. Data Analysis

The MaxQuant software (version 2.3.1.0) was used to process and quantify the obtained data [52]. The false discovery rate (FDR) was set to 1% for both proteins and peptides. A length of seven amino acids was specified as the minimum peptide length. The Andromeda search engine was used for the MS/MS spectra search against the AmoebaDB-65_AcastellaniiNeff_AnnotatedProteins database (downloaded from https://amoebadb.org/ on 1 June 2023, containing 14,979 entries). Enzyme specificity was set as C-terminal Arg and Lys, also allowing cleavage at proline bonds and a maximum of two missed cleavages. Dithiomethylation of cysteine was chosen as fixed modification, and N-terminal protein acetylation and methionine oxidation as variable modifications. The “match between runs” feature of MaxQuant was used both to transfer the identifications to other LC-MS/MS runs based on their masses and retention time (maximum deviation 0.7 min) and for quantification. Quantifications were achieved using the label-free algorithm in MaxQuant [53]. Perseus 1.6.15.0 software was used to analyze the data [54].

### 2.9. Evaluation of Nitroxoline Mechanism of Action

Apoptosis (programmed cell dead) and necrosis (pathological cell death) are the two main described models of cell death. Apoptosis is characterized by the formation of protrusions on the surface of the plasma membrane with cell content, loss of cell volume, chromatin condensation, and fragmentation of the nucleus and chromosomal DNA. Necrosis, on the other hand, is characterized by irreversible morphological changes in the nucleus and cytoplasm that induce the host inflammatory response [55,56]. In this sense, the development of apoptotic death in parasites could prevent a toxic effect in host cells. In this study, we evaluated different possible mechanisms of action and cellular targets of nitroxoline in trophozoites of the *Acanthamoeba culbertsoni* strain. Amoebae were observed (100× magnification) using the inverted-light microscope EVOS™ FL Cell Imaging System M5000 (Life Technologies, USA). Furthermore, fluorescence quantification of all images (40× magnification) obtained was performed using Fiji software (*Fiji is Just* ImageJ 1.53 q, National Institute of Health, Rockville Pike, MD, USA). All experiments were performed in triplicate.

#### 2.9.1. Analysis of Mitochondrial Function Disruption

Mitochondrial damage in *A. culbertsoni* trophozoites incubated with the IC_90_ of nitroxoline was evaluated using the JC-1 mitochondrial membrane potential (Δ*Ψm*) detection kit (Cayman Chemicals, Vitro SA, Madrid, Spain). The method used in this assay was based on a previous study [46].

#### 2.9.2. Measurement of ATP Production

The CellTiter-Glo Luminescent Cell Viability Assay (PROMEGA BIOTECH IBÉRICA S.L, Madrid, Spain) was used for the measurement of ATP levels in the trophozoites of *Acanthamoeba culbertsoni*. The effect of nitroxoline on ATP production was evaluated by incubating a determined concentration of trophozoites (10^5^ cells/mL) in PYG medium with the previously calculated IC_90_ for 24 h at 26 °C.

#### 2.9.3. Chromatin Condensation Detection

To assess if *A. culbertsoni* trophozoites incubated with the IC_90_ of nitroxoline underwent programmed cell death, the Hoechst 33342/PI (Life Technologies, Madrid, Spain) apoptosis detection kit was used in this study. The experiment was carried out by following a protocol previously defined [46].

#### 2.9.4. Plasma Membrane Permeability

The SYTOX™ Green reagent (Life Technologies, Madrid, Spain) was used to detect alterations in plasma membrane permeability in trophozoites incubated with the previously calculated IC_90_ value of nitroxoline [46].

#### 2.9.5. Evaluation of Intracellular ROS Production

The CellROX^®^ Deep Red fluorescent probe (Life Technologies, Madrid, Spain) was used to detect the production of intracellular reactive oxygen species (ROS) in trophozoites treated with the IC_90_ of nitroxoline [46].

### 2.10. Statistical Analysis

In all experiments, the data are expressed as mean ± standard deviation of at least three independent experiments. In order to highlight the effects of nitroxoline on all strains of *Acanthamoeba*, a statistical comparison was conducted using two-way analysis of variance (ANOVA). Moreover, to highlight the effect of nitroxoline, the measurements of mean fluorescence intensity obtained from different assays for *Acanthamoeba culbertsoni* trophozoites were statistically compared by one-way analysis of variance (ANOVA); a *p*-value (*p*) < 0.05 denoted the presence of a statistically significant difference. The statistical analyses were carried out using Sigma Plot 12.0 statistical analysis software (Systat Software, Düsseldorf, Germany ), and the graphs were obtained using GraphPad Prism 9.0. program (GraphPad Software, San Diego, CA, USA).

## 3. Results

### 3.1. In Vitro Activity of Nitroxoline against Trophozoites and Cysts of Acanthamoeba *spp.*

In the present study, nitroxoline was tested for its activity against six environmental and clinical *Acanthamoeba* strains. The determined IC_50_ values against both trophozoites and cysts are presented in Table 1.

According to Table 1, nitroxoline was effective in inhibiting all strains, with an IC_50_ ≤ 3.24 ± 0.56 µM for the trophozoite stage and an IC_50_ ≤ 0.98 ± 0.23 µM for the cyst stage. Two-way ANOVA analysis revealed that both the trophocidal and the cysticidal activities were significantly affected by the type of strain and the drug used, with *p* < 0.001. Focusing on the drug effect, we could conclude that the less effective drug was chlorhexidine, while the difference in activity for nitroxoline and voriconazole was generally non-significant. Whereas *A. culbertsoni* was the most resistant strain to voriconazole in the trophozoite stage, *A. quina* and *A*. L-10 were the most resistant strains towards both nitroxoline and chlorhexidine. As for the cyst stage, we found that *A. polyphaga* was the most resistant strain to chlorhexidine and voriconazole, while *A. culbertsoni* was the most resistant strain to nitroxoline.

#### Evaluation of Nitroxoline Effect on Cellular Events

The evaluation of nitroxoline effects on various cellular events was conducted on *A. culbertsoni* treated with the IC_90_ (5.31 ± 0.98 µM) for 24 h.

### 3.2. Nitroxoline Affects Both Cellular Morphology and Cytoskeleton Structure in Fixed Cells

**Figure 2:**
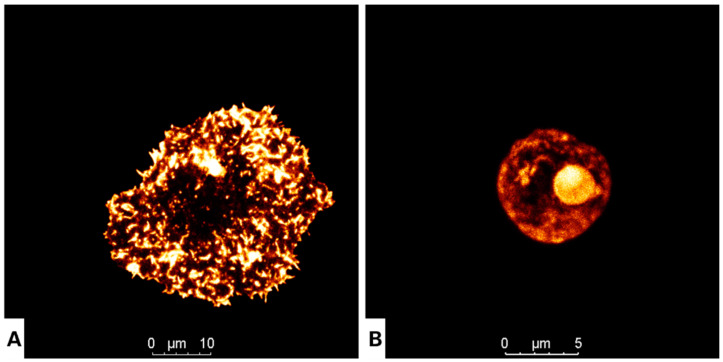
Evaluation of the effect of nitroxoline at the IC_90_ on trophozoites of *Acanthamoeba culbertsoni* (**B**; scale bar 5, µm) for 24 h. Phalloidin-TRITC staining of the polymerized actin cytoskeleton, showing the normal organization of the actin network, with an orange fluorescence in negative control cells (**A**; scale bar, 10 µm). All images (63×) were obtained using the inverted-light confocal microscope Leica DMI 4000 B.

**Figure 3:**
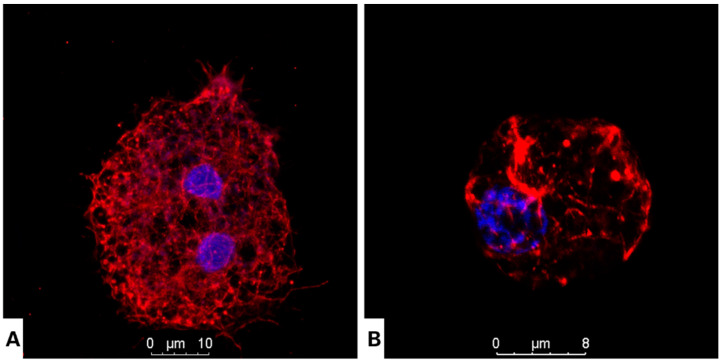
Intracellular organization of tubulin microtubules visualized using anti-tubulin antibodies emitting a red fluorescence. Trophozoites of *Acanthamoeba culbertsoni* incubated with the IC_90_ of nitroxoline for 24 h, showing disorganization or destruction of the tubulin microtubules (**B**; scale bar, 8 µm). Tubulin microtubules demonstrated a normal conformation in control cells (**A**; scale bar, 10 µm). Mounting DAPI solution for DNA staining shows a blue fluorescence. Images (63×) were obtained by using the inverted-light confocal microscope Leica DMI 4000 B.

Two proteins were chosen to determine the effect of nitroxoline on the cytoskeleton of *Acanthamoeba*: actin and tubulin. The analysis was conducted using confocal fluorescent microscopy. The actin network is considered an important target to inhibit *Acanthamoeba*, as it is a crucial element in the adhesion, motility and pathogenicity of this protozoon. The phalloidin-TRITC dye was used to stain the actin network. The treated cells showed a dramatic morphological change accompanied by a remarkable loss of cell volume compared to the negative control (Figure 2). As for tubulin, the damage caused by nitroxoline was assessed using an indirect immunofluorescence assay. After 24 h of treatment, the cells showed similar features as those previously observed, with a highly reduced cell size and uniformly distributed microtubules, while the untreated cells revealed a well-defined microtubule network throughout their cytoplasm (Figure 3).

### 3.3. Visualization of Autophagic Vacuoles in A. culbertsoni Using the Dye Monodansylcadaverine

**Figure 4:**
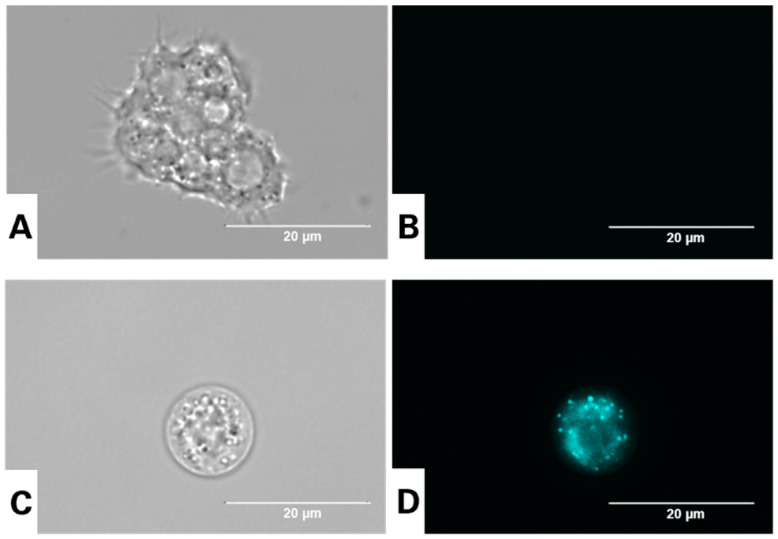
Evaluation of the presence of autophagic vacuoles in *Acanthamoeba culbertsoni* trophozoites incubated with the IC_90_ of nitroxoline for 24 h using the dye monodansylcadaverine (**C**,**D**). Autophagic vacuoles are indicated by light blue fluorescence. Negative control cells (**A**,**B**). The trophozoites (100×) were observed using the EVOS™ FL Cell Imaging System M5000 (scale bar, 20 µm).

Monodansylcadaverine is one of the main specific dyes to stain autophagic vacuoles. Endowed with autofluorescence, MDC can interact with lipids, thus accumulating in hydrophobic vacuoles and emitting higher fluorescence from these structures than from other cell compartments. Various reports confirmed the accumulation of MDC in the perinuclear area. The results shown in Figure 4 revealed the presence of various punctate spheric structures distributed in the cytoplasm in treated cells [57,58].

### 3.4. Nitroxoline Effect on the Proteomic Profile of Acanthamoeba Castellanii L-10

**Figure 5:**
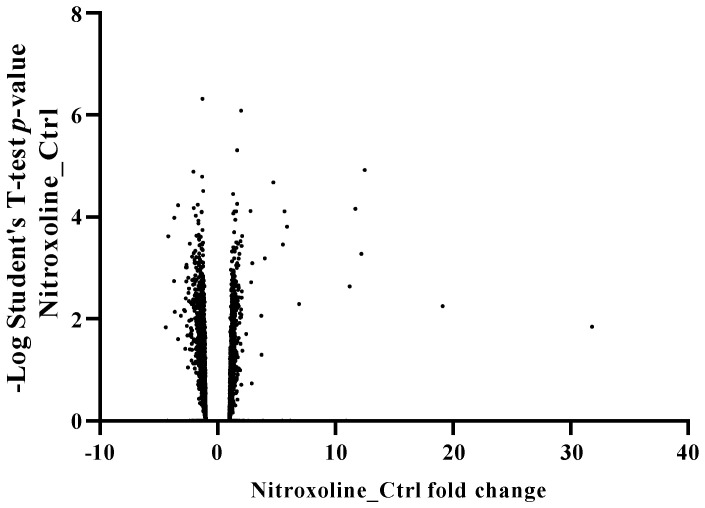
Volcano graph expressing the logarithmic Student’s *t*−test *p*−value as a function of protein expression fold change between nitroxoline-treated and control cells.

To elucidate the processes associated with nitroxoline chemotherapy, we opted to employ a whole-cell label-free proteomic approach. Two groups of *Acanthamoeba castellanii* L-10 parasites in the trophozoite stage were prepared: untreated cells and trophozoites treated with nitroxoline at the IC_50_. Proteins were extracted from each culture after 24 h of treatment. The proteomic profiling of both cultures resulted in the identification of 4565 proteins (Supplementary Materials, proteomic analysis). The proteomic analysis revealed that a total of 95 proteins were differentially expressed at least twofold compared to the negative control after nitroxoline exposure; among these, 60 and 35 proteins were downregulated and upregulated, respectively (Figure 5).

### 3.5. Nitroxoline Causse Mitochondrial Dysfunction in A. culbertsoni

Normally, healthy cells have stable levels of intracellular ATP, Δ*Ψm* and ROS, which vary slightly depending on various physiological conditions. Hence, their constant and/or very large variations could trigger a degenerative process [59].

#### 3.5.1. Nitroxoline Prevents the Aggregation of the JC-1 Dye

**Figure 6:**
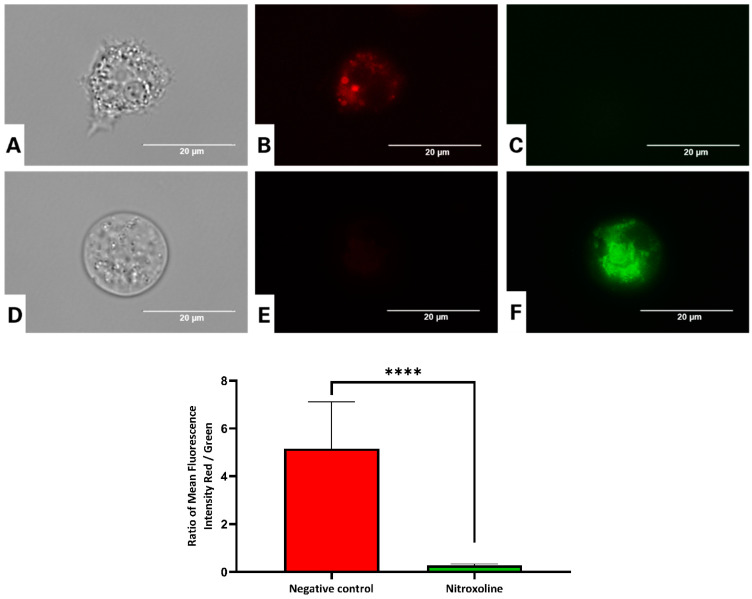
Use of the JC-1 dye to evaluate the collapse of the mitochondrial membrane potential in *Acanthamoeba culbertsoni* trophozoites incubated with the IC_90_ of nitroxoline (**D**–**F**) for 24 h. Control cells (**A**–**C**). All images (100×) were obtained using the inverted-light microscope EVOS™ FL Cell Imaging System M5000 (scale bar, 20 µm). Data showed in the graph are presented as means ± SD, **** *p* < 0.0001; the results demonstrated significant differences when comparing cells treated with nitroxoline to negative control cells. Differences between the mean values of the fluorescence intensity red/fluorescence intensity green ratio were assessed using one-way analysis of variance (ANOVA).

The JC-1 dye was employed to investigate the effect of the antibiotic on Δ*Ψm*. The assay is based on the accumulation of the potential-dependent dye in healthy mitochondria and on its emission of red fluorescence. After 24 h of incubation, we could appreciate an increase in green fluorescence, indicating the dye remained in the cytoplasm as a monomer. A statistical analysis confirmed that both red and green fluorescence intensities were significantly different between treated and untreated cells. The graph presented in Figure 6 shows a decrease in the red/green fluorescence ratio in treated cells, indicating an augmentation in green fluorescence (Figure 6 and Figure S1).

#### 3.5.2. Nitroxoline Induces a Decrease in the ATP Levels

**Figure 7:**
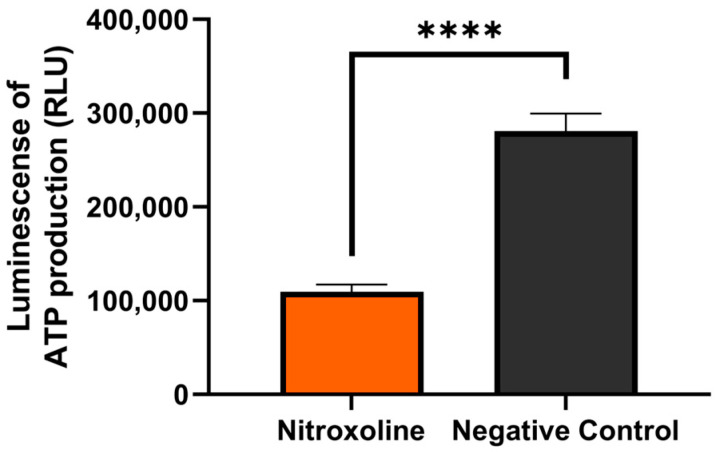
Luminescence indicating ATP production in trophozoites of *Acanthamoeba culbertsoni* after incubation with the IC_90_ of nitroxoline for 24 h using the CellTiter-Glo^®^ luminescent cell viability assay. Nitroxoline significantly decreased the level of mitochondrial ATP production in trophozoites of *A. culbertsoni* compared to the negative control, *p <* 0.0001 (****).

Generally, mitochondria dysfunction leads to a decrease in cellular energy (ATP) production. In the present study, we measured the production of ATP using the CellTiter-Glo^®^ reagent. One-way analysis variance (ANOVA) was carried out to test the statistical differences between the means. The trophozoites treated with nitroxoline showed a highly significant decrease in ATP levels compared to the negative control, *p <* 0.0001 (****) (Figure 7). After 24 h of incubation, the intracellular ATP level dropped in the treated cells to 41% compared to the negative control.

### 3.6. In Vitro Determination of the Cell Death Mode Induced by the Nitroxoline

#### Analyzing Cell Death by Double Nuclear Staining with Hoechst 33342/PI

**Figure 8:**
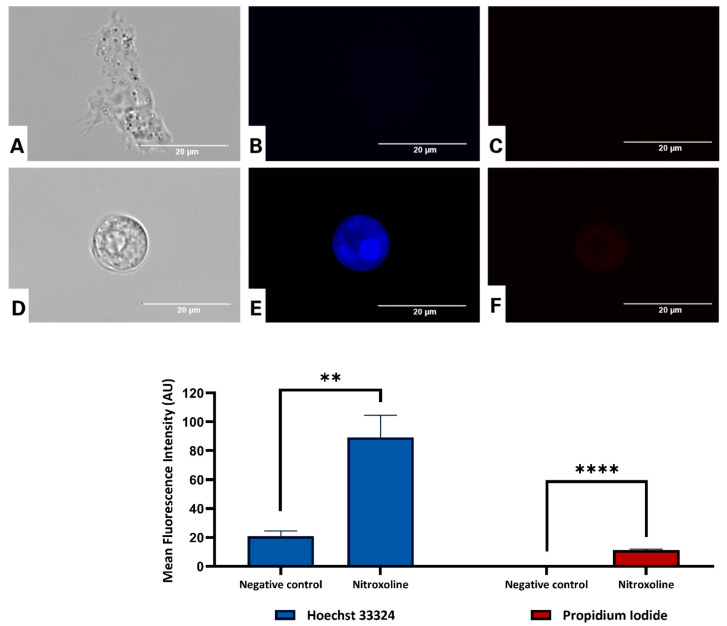
Effect of the IC_90_ of nitroxoline in trophozoites of *Acanthamoeba culbertsoni* (**D**–**F**) determined using a Hoechst 33342/PI apoptosis detection kit, after incubation of the cells for 24 h. Negative control (**A**–**C**). Treated trophozoites revealed bright blue-fluorescent nuclei, evidencing chromatin condensation (programmed cell death). The inverted-light microscope EVOS™ FL Cell Imaging System M5000 was used to analyze the images (100×, scale bar, 20 µm). The graph represents the mean fluorescence intensity (AU) of cells stained with the Hoechst 33324/PI kit. Data are presented as means ± SD, ** *p* < 0.01 and **** *p* < 0.0001; the results showed significant differences between cells treated with nitroxoline and negative control cells.

The Hoechst 33258 dye is often used to visualize the nuclear changes and apoptotic body formation that distinguish apoptotic cells from necrotic and healthy ones. As revealed in Figure 8, the treated cells, mainly their nuclear zone, emitted bright blue fluorescence. A statistical analysis of the mean fluorescence confirmed that nitroxoline increased by four times the blue fluorescence intensity compared to the negative control. In addition, since propidium iodide is not permeant to healthy cells, it is also commonly used to detect dead cells in a population. In this study, propidium iodide did stain amoebae in conjunction with Hoechst 33258, suggesting a late apoptotic stage (Figure 8 and Figure S2).

### 3.7. Nitroxoline Could Alter Plasma Membrane Permeability in Treated Cells

**Figure 9:**
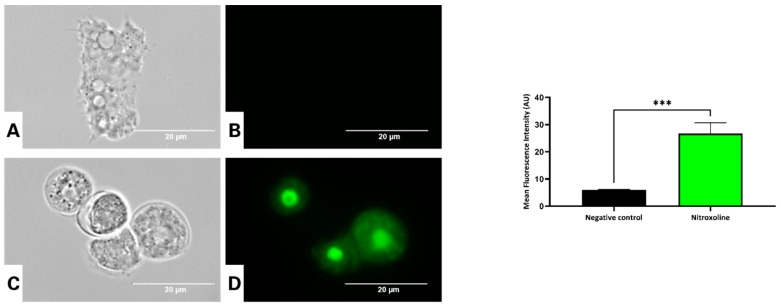
Evaluation of membrane permeability in trophozoites of *Acanthamoeba culbertsoni* incubated with the IC_90_ of nitroxoline (**C**,**D**) compared with the negative control (**A**,**B**), using the SYTOX™ Green reagent after 24 h of incubation. Trophozoites with alterations in plasma membrane permeability showed an intense green fluorescence in the nucleus. The inverted-light microscope EVOS™ FL Cell Imaging System M5000 was used to obtain all images (100×, scale bar, 20 µm). The graph shows the mean fluorescence intensity (AU) emitted by the stained cells. Data are presented as means ± SD, *** *p* < 0.001; the results showed significant differences between cells treated with nitroxoline and negative control cells.

Fluorescence microscopy was used to evaluate the possible effects of the antibiotic nitroxoline on cell membrane permeability, using the fluorescent probe SYTOX™ Green. This high-affinity DNA stain penetrates cells with compromised plasma membranes. The intensity of this dye enhances 500-fold upon nucleic acid binding. Our data showed that nitroxoline could increase plasma membrane permeability in treated cells (Figure 9 and Figure S3). This observation was confirmed by ANOVA, which indicated that the treated cells emitted significantly higher green fluorescence than the negative control (Figure 9).

### 3.8. Nitroxoline Increases the Cytosolic Level of Reactive Oxygen Species (ROS) in A. culbertsoni

**Figure 10:**
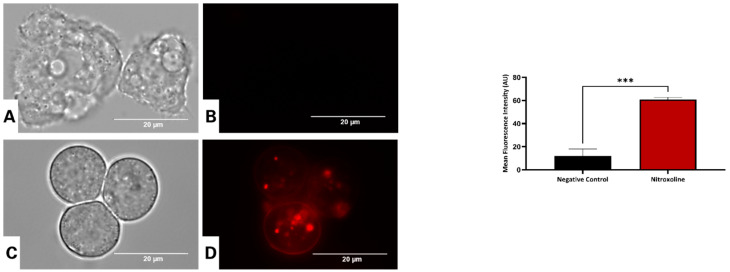
Evaluation of reactive oxygen species (ROS) production using the CellROX^®^ Deep Red fluorescent probe in *Acanthamoeba culbertsoni* trophozoites, incubated with the IC_90_ of nitroxoline (**C**,**D**) for 24 h. Control cells (**A**,**B**). All images (100×) were obtained using the inverted-light microscope EVOS™ FL Cell Imaging System M5000 (scale bar, 20 µm). Data shown in the graph are presented as means ± SD, *** *p* < 0.001; the results demonstrated significant differences between cells treated with nitroxoline and negative control cells.

The collapse of the mitochondria membrane potential after 24 h could be the results of the accumulation of reactive oxygen species (ROS) in the mitochondria. In the present work, the ROS levels were detected by fluorescence microscopy using the CellROX^®^ Deep Red dye. Figure 10 and Figure S4 illustrate the increase in ROS levels in the cytosol. One-way ANOVA was conducted, and the mean fluorescence emitted by the treated cells was higher and significantly different from that of the negative control (Figure 10).

## 4. Discussion

Nitroxoline is an antimicrobial that belong to the group of hydroxyquinoline derivates [60]. Bergogne-Berezin et al. (1987) reported its use in the treatment of urinary infections, particularly those caused by *Escherichia coli* [61]. In Germany, nitroxoline is recommended among other oral antimicrobials as first-line therapy against uncomplicated urinary tract infections (UTIs) [28,30]. Recently, several authors ascertained other pharmacological properties of this old antibiotic, such as its antifungal, amoebicidal and anticancer properties [31,36,39,43].

The main objective of the present work was to corroborate the activity of nitroxoline against *Acanthamoeba* spp. and to propose its repurposing to manage infections caused by *Acanthamoeba*. The activity of nitroxoline was assessed in vitro against the trophozoites and cysts of six different *Acanthamoeba* strains. The activity was strain-dependent: while *A. castellanii* Neff was the most sensitive strain, *A. castellanii* L-10 and *A. culbertsoni* were among the most resistant strains. Nevertheless, the antibiotic was able to inhibit both stages of the studied strains. While nitroxoline exhibited the same activity as voriconazole, both drugs were much more potent in inhibiting the parasite than chlorhexidine. The present study reaffirms the amoebicidal activity of nitroxoline in addition to its reported activity against two other pathogenic free-living amoebae, i.e., *Balamuthia mandrillaris* [31] and *Naegleria fowleri* [43].

After oral administration, the systemic concentrations of nitroxoline are considered to be low, since nitroxoline is rapidly conjugated in the liver and excreted in the urine, which explains its use to treat UTIs [60,62]. Although some authors described the presence of amoebae in the context of urinary tract infection [13], the clinical relevance of amoebae for UTIs remains questionable. Whether nitroxoline achieves relevant concentrations in other body compartments besides the urinary tract, such as the eye or the central nervous system, has never been systematically investigated, and the available results are only based on some in vivo reports [40,42,60]. Therefore, the potential of nitroxoline to treat amoeba infections should not be overestimated based on in vitro findings, and more research is needed. However, its in vitro amoebicidal activity against *Acanthamoeba* spp. demonstrated in this study can be considered excellent and could lead to further research with respect to its topical administration in the context of keratitis treatment. The fact that nitroxoline is already approved, with a recently confirmed safety profile [28], may facilitate clinical trial development or even its compassionate use, as previously reported for a *Balamuthia mandrillaris* encephalitis [42]. Also, in the future, nanoparticle-based approaches, which have been extensively investigated for both *Acanthamoeba* [47,63] and nitroxoline [64], may help to distribute nitroxoline to specific body compartments with targeted drug delivery.

The mode of action of nitroxoline as an antimicrobial and anticancer agent is still ambiguous. Various studies were conducted to elucidate its mechanism of action. In cancer cells, Sup Shim et al. (2010) showed the ability of nitroxoline to inhibit angiogenesis and, subsequently, the growth of various cancers including breast and human bladder cancer [65]. In addition, Jelena Lazovic et al. (2015) outlined the growth inhibition of glioblastoma cells induced by nitroxoline in vitro and in vivo in a mouse model [40]. Moreover, they described this antitumoral effect as the result of cell cycle arrest in the G1/G0 phase and the induction of apoptosis via caspase-3 and cleaved poly(ADP-ribose) polymerase. In the present study, we investigated the programmed cell death induced by nitroxoline in *Acanthamoeba culbertsoni* by evaluating different metabolic events. In treated cells, nitroxoline could induce several physiological features matching with the apoptotic and autophagic processes, namely, chromatin condensation, formation of autophagic vacuoles and mitochondrial dysfunction. Chang et al. (2015) demonstrated that nitroxoline could evoke simultaneously apoptosis and autophagy in prostate cancer by the regulation of the AMPK/mTOR signaling pathway [66].

To improve our understanding of how nitroxoline inhibits the studied parasite, a proteomic profile analysis was conducted on treated and untreated *Acanthamoeba castellanii* L-10. The most downregulated proteins, specifically enrolled in metabolic and biosynthesis pathways, were an amidohydrolase superfamily protein; a diacylglycerylN,N,N-trimethylhomoserine synthesis protein; a glucosyl hydrolase family protein; and dihydrothymine dehydrogenase, among others. Interestingly, we observed that nitroxoline could inhibit the sterol C22 desaturase-like protein, which was reported by Thomson et al. (2017) as a drug target candidate [67]. Ergosterol is the major sterol in *Acanthamoeba* spp. Inhibiting the biosynthesis of this sterol could therefore inhibit the growth and encystation of the parasite. In several reports, the antimicrobial activity of nitroxoline based on chelating and sequestering biologically important divalent metal ions such as Mg^2+^, Mn^2+^, Fe^2+^ and Zn^2+^, was confirmed [68,69,70], indicating that nitroxoline could induce the deregulation of molecules involved in cell bioenergetics, leading to a collapse of the mitochondrial membrane potential. In the present work, various oxidoreductase and electron transport chain proteins were inhibited, namely, cytochrome b5 heme-binding domain-containing protein and Rieske domain-containing protein [69,71]. On the other hand, metal ion chelation by nitroxoline was found to inhibit the function of bacterial RNA polymerase as well as biofilm formation by multiple pathogens and reduce bacterial adhesion to bladder epithelial cells and catheters [35,72]. Apart from those effects, nitroxoline was described to inhibit the expression of the cysteine proteinase cathepsin B in cancer cells. In *Acanthamoeba* spp., this protein is involved in numerous functions such as nutrition, development, encystation and pathogenicity [73]. Our proteomic analysis revealed that the cysteine proteinase was downregulated in treated cells compared to the negative control. Inhibiting this cysteine proteinase could inhibit the encystation process in various protozoan parasites such *Acanthamoeba* spp. and *Entamoeba invadens* [74].

Among the proteins upregulated in treated cells, we observed SHSP domain-containing proteins, demonstrating that the cells were under diverse environmental stresses. In fact, SHSPs play a crucial role in preventing apoptosis and maintaining cytoskeleton integrity [75]. In *Acanthamoeba* spp., these chaperone proteins play an imperative role in the defense system and virulence. When treated with nitroxoline at the IC_50_, the amoeba cells responded by activating their defense system including SHSPs and other proteins such as an O-methyltransferase family protein. This protein has been reported in plants as regulating a defense mechanism against environmental stress and infectious diseases [76]. During the present study, we observed that nitroxoline at the IC_90_ damaged the mitochondrial function, while at the IC_50_ it could activate the mitochondrial genome maintenance protein, as a response to the increase in ROS production. In this context, Hsin-Chen and Yau-Huei (2005) indicated in their study that the mitochondrial DNA content increases with ROS accumulation [77].

## 5. Conclusions

In this study, the antibiotic nitroxoline exhibited amoebicidal activity against six strains of *Acanthamoeba: A. castellanii* Neff, *A. polyphaga*, *A. griffini*, *A. quina*, *A. culbertsoni* and *A. castellanii* L-10. We demonstrated that nitroxoline induced features compatible with apoptosis and autophagy in trophozoites of *A. culbertsoni*, revealing chromatin condensation, mitochondrial damage, oxidative stress induced by ROS production and the formation of autophagic vacuoles. Nitroxoline altered the cytoskeleton of *Acanthamoeba*, inducing disorganization of the actin and tubulin networks. In addition, the most downregulated proteins in the treated trophozoites were the diacylglycerylN,N,N-trimethylhomoserine synthesis protein and dihydrothymine dehydrogenase, specifically enrolled in metabolic and biosynthesis pathways. The overexpression of SHSP domain-containing proteins demonstrated that the trophozoites of *Acanthamoeba* treated with nitroxoline were subjected to diverse environmental stresses, activating apoptotic or cellular defense response process. Finally, taking all the results into account, the antibiotic nitroxoline could be an excellent candidate for drug repurposing for the treatment of *Acanthamoeba* infections.

## Figures and Tables

**Table 1:** In vitro amoebicidal effect of nitroxoline against the trophozoite and cyst stages of different strains of *Acanthamoeba* spp.

*Acanthamoeba* Strains	Nitroxoline IC_50_ (µM)		Chlorhexidine IC_50_ (µM)		Voriconazole IC_50_ (µM)	
	Trophozoites	Cysts	Trophozoites	Cysts	Trophozoites	Cysts
***A. castellanii* Neff**	0.87 ± 0.19 ^aA^	0.28 ± 0.12 ^aA^	3.02 ± 0.89 ^aB^	5.97 ± 1.76 ^cC^	0.99 ± 0.04 ^aA^	3.45 ± 0.17 ^bB^
***A. polyphaga***	0.95 ± 0.01 ^aA^	0.81 ± 0.03 ^aA^	5.59 ± 0.04 ^bB^	9.41 ± 0.16 ^dC^	1.07 ± 0.02 ^aA^	6.98 ± 0.05 ^cB^
***A. griffini***	0.69 ± 0.01 ^aA^	0.84 ± 0.01 ^aA^	5.60 ± 0.07 ^bB^	7.38 ± 1.94 ^cB^	0.32 ± 0.01 ^aA^	0.92 ± 0.06 ^aA^
***A. quina***	3.24 ± 0.56 ^bB^	0.31 ± 0.05 ^aA^	5.31 ± 0.48 ^bCB^	4.04 ± 0.48 ^b^	0.54 ± 0.01 ^aA^	4.69 ± 0.09 ^bB^
***A.* L-10**	2.85 ± 0.58 ^bB^	0.11 ± 0.03 ^aA^	9.11 ± 0.29 ^dC^	1.30 ± 0.36 ^aA^	1.77 ± 0.15 ^abA^	0.51 ± 0.10 ^aA^
***A. culbertsoni***	1.17 ± 0.09 ^aA^	0.98 ± 0.23 ^aA^	8.11 ± 0.17 ^cC^	2.92 ± 0.28 ^bB^	1.93 ± 0.09 ^bB^	1.24 ± 0.15 ^aA^

Chlorhexidine and voriconazole were chosen as positive controls. Means for individual strains with different lowercase letters (a–d) are significantly different (*p* < 0.05). Means for individual compounds with different uppercase letters (A–C) are significantly different (*p* < 0.05).

## Data Availability

Data are contained within the article.

## Supplementary Materials

The following supporting information can be downloaded at: https://www.mdpi.com/article/10.3390/antiox12122081/s1, Figures S1–S4: Low magnification (40×) of the images in Figures 6, 8, 9 and 10. Second archive contains the proteomic analysis of trophozoites treated with nitroxoline.
