# Supplementary material for: Induction of Programmed Cell Death in Acanthamoeba culbertsoni by the Repurposed Compound Nitroxoline
Source: Antioxidants (Basel). 2023 Dec 6;12(12):2081. doi: 10.3390/antiox12122081 (PMC10740438; doi:10.3390/antiox12122081)
Supplement: Supplementary file 1 [file antioxidants-12-02081-s001.zip › Supplementary Material.pdf]

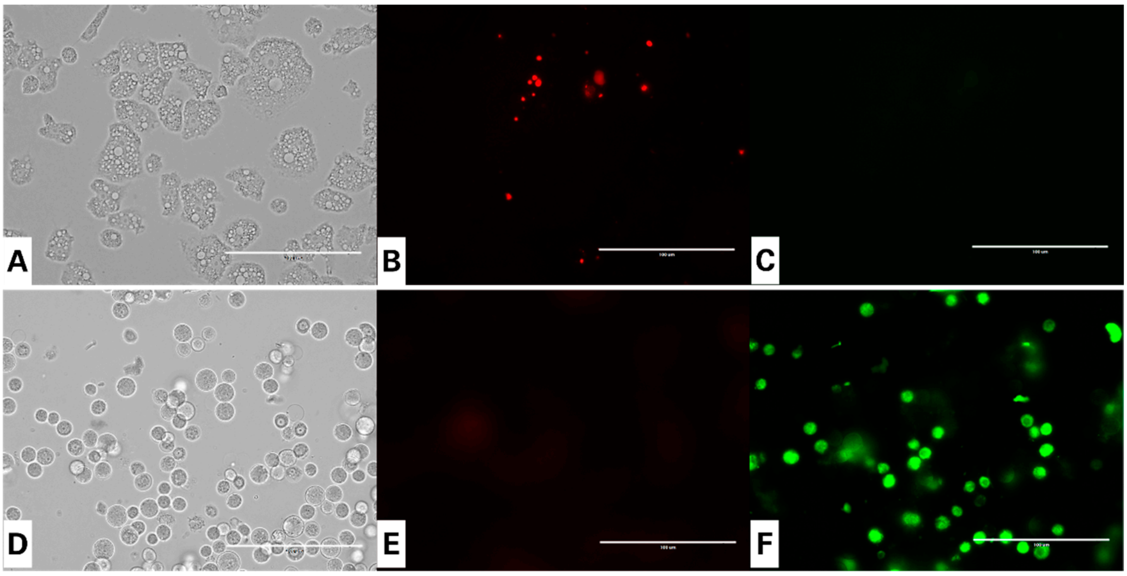

Figure S1. Lower magnification (40x) of the **Figure 6**. Images are based on EVOS™ FL Cell Imaging System M5000 (Scale Bar 100 µm).

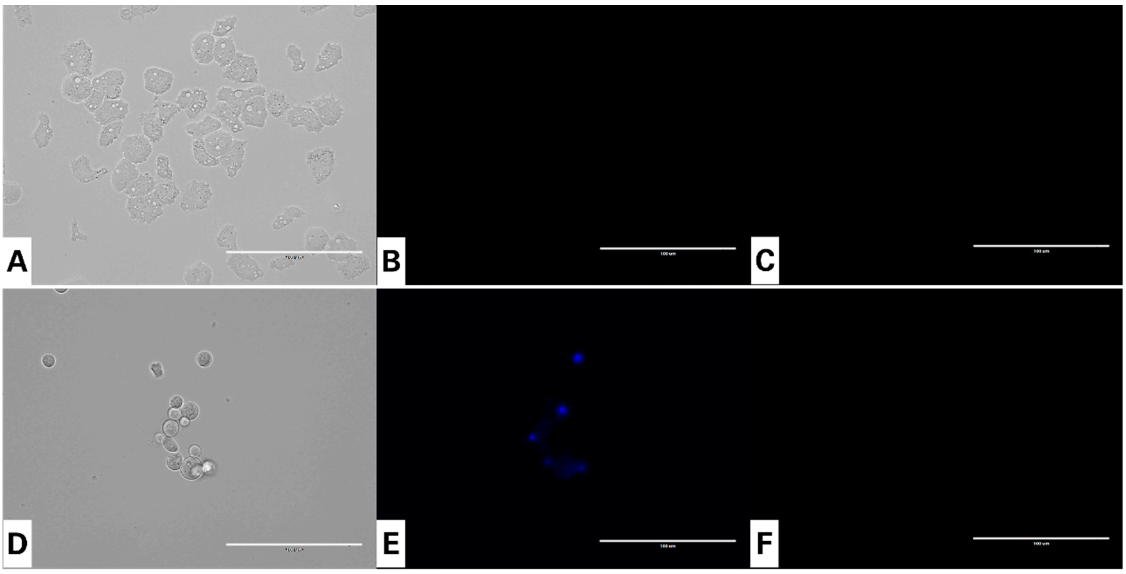

Figure S2. Lower magnification (40x) of the **Figure 8**. Images are based on EVOS™ FL Cell Imaging System M5000 (Scale Bar 100 µm).

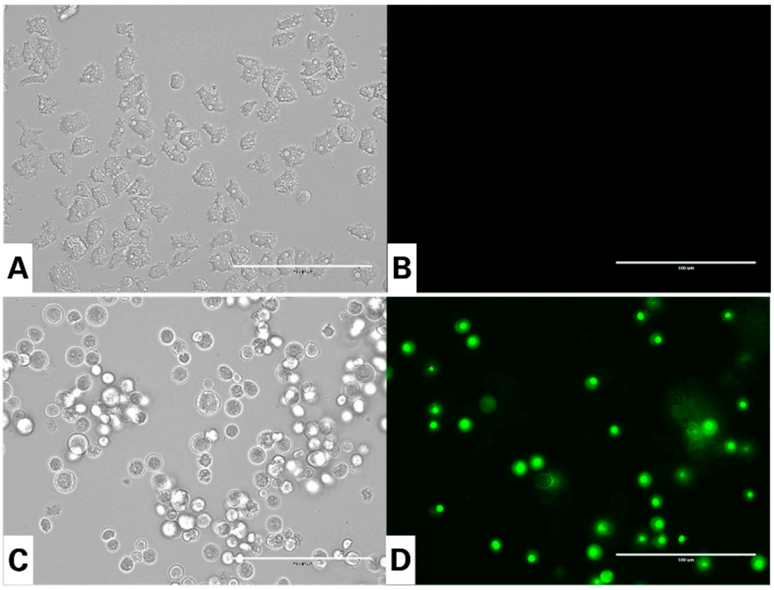

Figure S3. Lower magnification (40x) of the **Figure 9**. Images are based on EVOS™ FL Cell Imaging System M5000 (Scale Bar 100 µm).

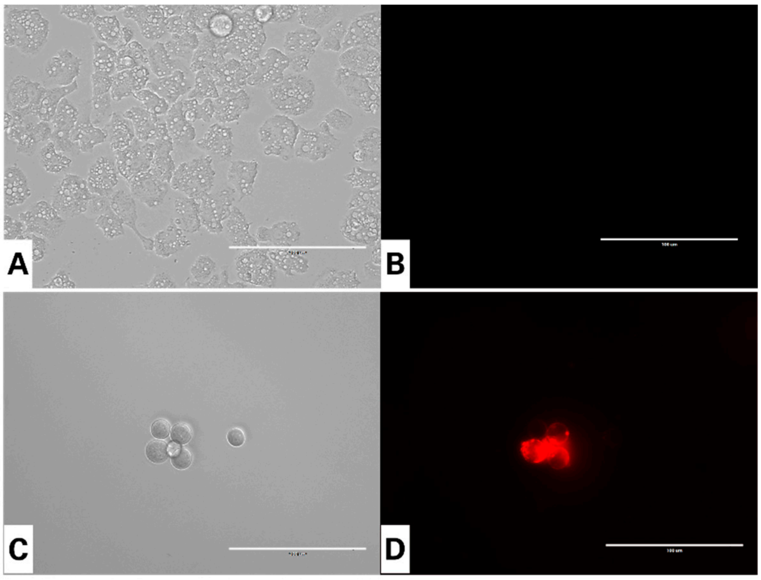

Figure S4. Lower magnification (40x) of the **Figure 10**. Images are based on EVOS™ FL Cell Imaging System M5000 (Scale Bar 100 µm).
